# Work intensity and quality of life can be restored following double-level osteotomy in varus knee osteoarthritis

**DOI:** 10.1007/s00167-022-06909-4

**Published:** 2022-03-10

**Authors:** Christoph Ihle, Julia Dorn, Atesch Ateschrang, Heiko Baumgartner, Moritz Herbst, Stefan Döbele, Tina Histing, Steffen Schröter, Marc-Daniel Ahrend

**Affiliations:** 1 Department of Traumatology and Reconstructive Surgery, BG Trauma Center Tübingen, Eberhard Karls University Tübingen, Tübingen, Germany; 2 Evangelisches Stift St. Martin Gemeinschaftsklinikum Mittelrhein, Koblenz, Germany; 3 Komitee Osteotomie der Deutschen Kniegesellschaft (DKG), Munich, Germany; 4 Department of Traumatology and Reconstructive Surgery, Diakonie Klinikum GmbH Jung-Stilling-Krankenhaus, Siegen, Germany; 5 AO Research Institute Davos, Davos, Switzerland

**Keywords:** Osteotomy, DLO, Double level osteotomy, Knee, Varus osteoarthritis, HTO, High tibial osteotomy, DFO, Distal femoral osteotomy

## Abstract

**Purpose:**

The purpose of this study was to assess changes in health-related quality of life (HRQL) and work intensity following double-level knee osteotomy (DLO). It was hypothesized that postoperative HRQL would be comparable to that of the general population and that work intensity can be restored in the short term.

**Methods:**

Twenty-four patients (28 varus knees; mechanical tibiofemoral angle: −11.0 ± 3.0° (−6.0 to −17.0), age: 49.1 ± 9.5 (31–65) years) who underwent DLO were included. The duration the patients were unable to work was evaluated. HRQL was measured with the SF-36 questionnaire, which consists of a physical (PCS) and mental component summary score (MCS). The pre- to postoperative changes in the PCS and MCS were analysed. The PCS and MCS were also compared to those of the general population, who has a reference score value of 50 points. The work intensity measured with the REFA classification and the Tegner activity scale were assessed preoperatively and at the final postoperative follow-up examination (18.0 ± 10.0 (5–43) months).

**Results:**

The duration that the patients were unable to work was 12.2 ± 4.4 (6–20) weeks. The PCS improved from 32.1 ± 11.3 (14.5–53.3) preoperatively to 54.6 ± 8.5 (25.2–63.7) (*p* < 0.001) at the final follow-up, and the MCS improved from 53.9 ± 11.1 (17.1–67.7) to 57.2 ± 3.1 (47.3–61.7) (n.s). The preoperative PCS was significantly lower than the reference score of the general population (*p* < 0.001), whereas the preoperative MCS was similar between the two groups (n.s*.*). At follow-up, no significant differences were observed between the PCS and the MCS of the patient group and those of the general population. Five patients who were unable to work prior to surgery due to knee symptoms returned to work with moderate (four patients) or even very heavy (one patient) workloads. The Tegner activity scale increased significantly from a median of 2.0 (0.0–5.0) to 4.0 (2.0–7.0) (*p* < 0.001).

**Conclusion:**

Our results demonstrate an improvement in quality of life and return to working activity following DLO in the short term. The HRQL can be improved by DLO in patients with varus knee osteoarthritis to the level of the general population. These results can assist surgeons in discussing realistic expectations when considering patients for DLO.

**Level of evidence:**

Study type: therapeutic, IV.

## Introduction

The health-related quality of life (HRQL) is an established method for measuring postoperative outcomes following knee surgery [21, 37]. The SF-36 [40] questionnaire determines the HRQL subjectively and provides a way to compare individual patients across age and sex differences with known scores of the German general population.

Knee osteoarthritis, chronic knee pain and loss of function can lead to limitations in daily activities [33]. They may also result in reduced work intensity or even require a change from jobs with high physical workloads to those with low workloads; additionally, heavy working conditions that involve frequent kneeling can also increase the risk of knee osteoarthritis [26]. Several nonsurgical and surgical procedures are available for treating osteoarthritis of the knee [6, 34]. In young and active patients with extra-articular varus malalignment and medial compartment osteoarthritis, high tibial osteotomy (HTO) is well established with good to excellent midterm outcomes and high rates of return to sporting activities [11, 23, 30]. Nevertheless, individual preoperative radiological analysis and surgical planning are mandatory for treatment selection. The indication and success of HTO depend on the localization (femoral or tibial) and the degree of the deformity [14, 32]. Especially for severe varus knee osteoarthritis, double-level osteotomy (DLO; open wedge HTO and closed wedge distal femoral osteotomy (DFO)) have received increased clinical and scientific attention in recent years [28, 39], and previous studies have reported significant improvements in various clinical outcomes [3, 28]. It has been shown that a large amount of correction in open wedge HTO with a resulting joint-line obliquity of 5° or more can induce high shear stress on articular cartilage [27]. Inferior results and additional varus deformity of the distal femur were found in patients after single open wedge HTO [41]. In these cases, DLO should be considered a treatment option [27]. To the authors’ knowledge, there is no study available describing the HRQL, the ability to return to work and changes in work intensity following DLO. Considering that adequate patient education prior to surgery is crucial and that unrealistic patient expectations have been shown to have a negative impact on patient-reported outcomes, this information would be of great importance in daily clinical practice [10, 12].

The aim of this retrospective study was to assess the change in HRQL and work intensity following DLO in patients with varus osteoarthritis. It is hypothesized that comparable HRQL values to those of the general population can be achieved after surgery and that work intensity can be restored in the short term.

## Materials and methods

**Fig. 1:**
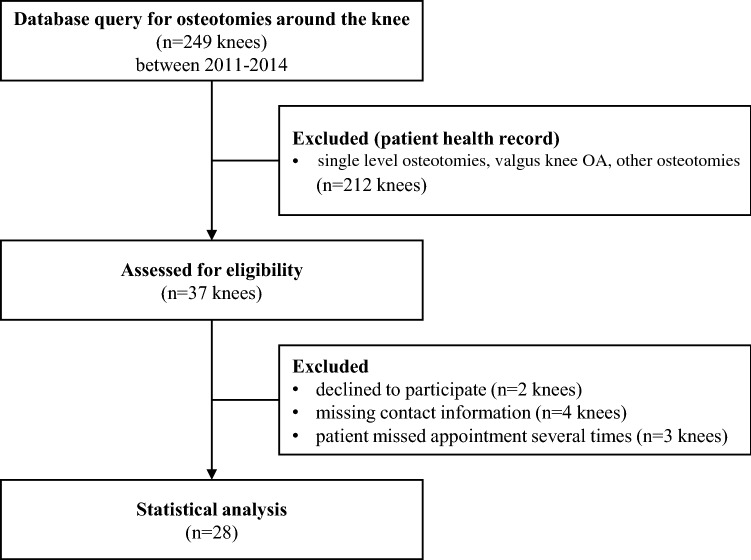
Patient flow chart shows patient acquisition and reasons for exclusion from the study

Within a 3-year timeframe, 249 patients underwent osteotomy for varus deformity of the lower extremity in one single traumatology and orthopaedic department. Of these, 33 patients (37 knees) were treated with DLO by two experienced surgeons. A clinical and radiological examination was performed before surgery and at follow-up at least 5 months following surgery (18.0 ± 10.0 (5–43) months). Nine patients declined to participate or were not available for the follow-up examination. At the final follow-up, data from 24 patients (28 knees) with a mean age of 49.1 ± 9.5 (31–65) years were available for further statistical analysis (Fig. 1). The demographic and radiological baseline data of the study population are shown in Table 1. Four patients received DLO on both knees; for these patients, there was an interval of at least 1 year between the first and second surgery, and the score was assessed for both sides at the same time after the second surgery. All scores, including the HRQL and the duration the patients were unable to work, were assessed for each knee. The study was approved by the local ethics committee (103/2013BO2). All participants gave their written informed consent to participate in the study.

**Table 1:** Patient baseline characteristics [Mean ± standard deviation (minimum–maximum)]

Follow-up [months]	18.0 ± 10.0 (5−43)
Gender	Male: 20 Female: 4
Age at time of surgery [years]	49.1 ± 9.5 (31–65)
Body mass index [kg/m^2^]	29.9 ± 4.9 (21−43)
Affected side	Left: 15 Right:13
mTFA preoperative [°]	−10.8 ± 2.8 (−6.0 to −17.0)
MPTA preoperative [°]	84.3 ± 2.7 (78.0–88.0)
mLDFA preoperative [°] JLCA preoperative [°] JLCA postoperative [°]	91.6 ± 2.3 (86.0–96.0) 3.5 ± 1.8 (0.0–7.0) 3.0 ± 1.9 (0.0–8.0)

*mTFA* mechanical tibiofemoral angle; *MPTA* medial proximal tibia angle, *mLDFA* mechanical lateral distal femur angle; *JLCA* joint line convergence angle; “−” means varus, “ + ” means valgus deviation of the mTFA

### Treatment with DLO

Deformity analysis and osteotomy planning were performed using long-leg weight-bearing radiographs at baseline and follow-up. The mediCAD^®^ digital planning software (mediCAD Hectec, Altdorf/Landshut, Germany) was used. Indication criteria for DLO included symptomatic varus malalignment (mechanical tibiofemoral angle (mTFA) greater than −5° varus) and medial compartment osteoarthritis; the procedure was performed when simulation of an open wedge HTO resulted in a medial proximal tibial angle (MPTA) of more than 94° or when deformity analysis showed a mechanical lateral distal femoral angle (mLDFA) of over 90° in combination with an MPTA under 87°. The surgical technique and radiological results have been published previously [39]. For rehabilitation, all patients were assigned to 20 kg partial weight-bearing using two crutches for 6 weeks followed by full weight-bearing. No braces or casts were used. Postoperatively, active physiotherapy was started after the removal of drainage. In addition, the patients used an active motion splint (CAMOped; OPED, Valley/Oberlaindern, Germany) for 6 weeks [39].

### Outcome parameters

The HRQL was measured using the SF-36 questionnaire, which consists of eight subscales: physical functioning (PF), role physical (RP), bodily pain (BP), general health (GH), vitality (VT), social functioning (SF), role emotional (RE) and mental health (MH). From these subscales, the mental component summary score (MCS) and the physical component summary score (PCS) can be calculated. All subscales, as well as the MCS and PCS, were adjusted for age and sex, which allows comparisons of the scores of individual patients with those of the general population for the same demographic characteristics [15]. Norm-based scoring was conducted in which the raw values were transformed such that values above 50 corresponded to a score above the general population, while a score below 50 corresponded to a score below the age- and sex-adjusted general population [15].

Duration of the inability to work was recorded. Work intensity was assessed by the REFA classification [38] and the Tegner activity scale preoperatively and at the short-term follow-up. The patient’s work intensity was assigned to one of four categories according to the REFA classification (REFA = 1: low work intensity; REFA = 2: moderate work intensity; REFA = 3: high work intensity; REFA = 4: very heavy work intensity); this classification system has been used to describe potential workload changes in orthopaedics [20, 38]. The REFA classification and the duration of the inability to work, were assessed only in patients who were able to work prior to the operation. In patients who were already retired, the work-related analyses were not performed. Furthermore, the visual analogue scale (VAS) score was determined to measure and compare pre- and postoperative knee pain.

### Statistical analysis

Statistical analysis was performed with IBM SPSS^®^ Version 24 (Armonk, NY, USA). Descriptive data are presented as the mean ± standard deviation (minimum–maximum) or the median (minimum–maximum). The normality of the distribution of the data was tested using the Shapiro–Wilk test. The Wilcoxon test was used to assess the pre- to postoperative differences in the SF-36 total and subscale scores, the VAS scores and the Tegner activity levels. To compare the total and subscale scores of the SF-36 to those of the general population, one-sample *t* tests were conducted using a score value 50 as the test value. The statistical significance level was set at *p* ≤ 0.05 for all tests.

## Results

**Fig. 2:**
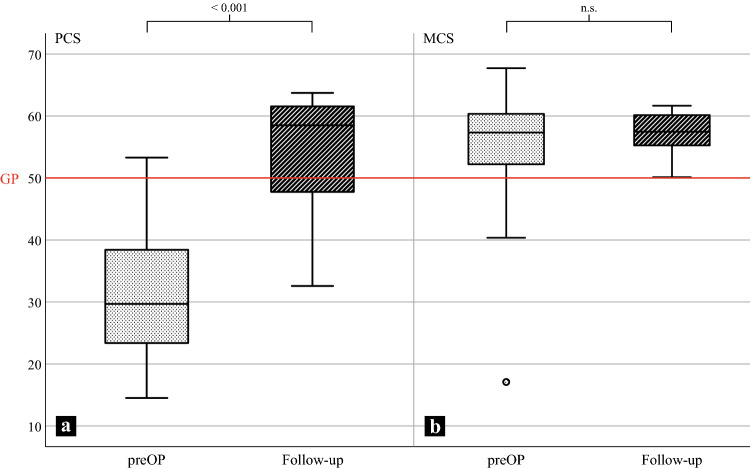
The physical health component summary scores (PCS) improved significantly from the preoperative (preOP) to the postoperative period (*p* < 0.001). The mental health component summary scores (MCS) did not change significantly between the two time points (n.s.). The reference score values for the general population were 50 after adjusting for age and sex (red line)

All SF-36 subscale scores improved following DLO in the short-term follow-up (Table 2). The PCS showed a significant improvement from 32.1 ± 11.3 (14.5–53.3) to 54.6 ± 8.5 (25.2–63.7) (*p* < 0.001) (Fig. 2a), while the increase in the MCS was not statistically significant (53.9 ± 11.1 (17.1–67.7) to 57.2 ± 3.1 (47.3–61.7), n.s.) (Fig. 2b).

**Table 2:** SF-36 with the subscales at both acquisition times

Score	Preoperative	Follow-up	*P* value
**PCS**	32.1 ± 11.3 (14.5–53.3)	54.6 ± 8.5 (32.6–63.7)	< 0.001
PF	29.3 ± 15.5 (−6.2–52.6)	49.7 ± 8.8 (26.7–58.2)	< 0.001
RP	33.4 ± 10.5 (17.4–53.2)	49.9 ± 8.8 (23.3–57.1)	< 0.001
BP	36.9 ± 7.6 (26.0–56.7)	57.9 ± 6.3 (45.4–64.4)	< 0.001
GH	53.8 ± 6.1 (34.4–62.2)	61.6 ± 4.7 (49.4–68.9)	< 0.001
**MCS**	53.9 ± 11.1 (17.1–67.7)	57.2 ± 3.1 (50.1–61.7)	n.s.
VT	46.8 ± 10.9 (25.3–61.3)	54.7 ± 5.2 (42.2–64.4)	0.001
SF	48.6 ± 15.3 (10.8–57.8)	56.2 ± 3.2 (42.2–57.8)	0.012
RE	45.3 ± 16.2 (−9.4–54.3)	53.5 ± 2.5 (42.4–55.3)	0.005
MH	52.8 ± 8.6 (33.6–63.9)	57.6 ± 4.3 (47.1–63.9)	0.003

The changes between preoperative and the follow-up regarding the two summary scores (PCS, MCS) and the subscales were tested using the Wilcoxon-test. PCS is formed by the subscales representing physical health, while MCS is based on the subscales representing mental health

All values are arithmetic means ± standard deviation (minimum−maximum)

All values are adjusted for age and sex differences

*PCS* physical health component summary score, *MCS* mental health component summary score, *PF* physical functioning, *RP* physical role functioning, *BP* bodily pain, *GH* general health perception, *VT* vitality, *SF* social role functioning, *RE* emotional role functioning, *MH* mental health

The preoperative scores for the subscales describing physical components (PF, RP, BP, GH) and the summary PCS showed significantly lower values than corresponding age- and sex-adjusted values of the general population (PF, RP, BP: *p* < 0.001; GH: *p* = 0.007). The differences between the patients and the general population regarding the preoperative MCS and the scores of the corresponding subscales (VT, SF, RE, MH) were not significantly different (all n.s.).

**Fig. 3:**
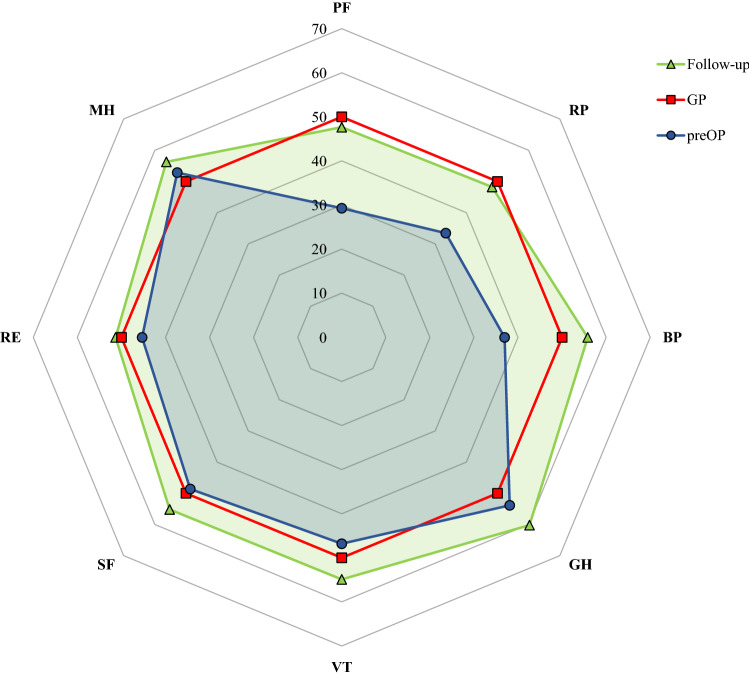
Radar plot showing the pre- and postoperative subscale scores of the SF-36 and a comparison with those of the general population. Preoperatively, the scores of the physical health subscales (PF, RP, BP) were lower than those of the general population (50 points). Postoperatively, the subscale scores increased and were similar to those of the general population. The preoperative and postoperative scores of the mental health subscales (MH, VT, SF, RE) were both similar to the scores of the general population (GP). *PF* physical functioning, *RP* physical role functioning, *BP* bodily pain, *GH* general health perception, *VT* vitality, *SF* social role functioning, *RE* emotional role functioning, *MH* mental health, *preOP* preoperative recording

At follow-up, the PCS and the PF and RP subscale scores were similar (n.s.), while the BP and GH subscale scores (*p* < 0.001) were greater than those of the general population*.* The MCS and the scores of the VT, SF and MH subscales were significantly higher than those of the general population at the time of follow-up (all *p* < 0.001). For RE, no significant difference was observed between the scores of the patient group and those of the general population (n.s.). The values of all subscales prior to surgery and during follow-up and the reference values of the general population are shown in Fig. 3.

**Fig. 4:**
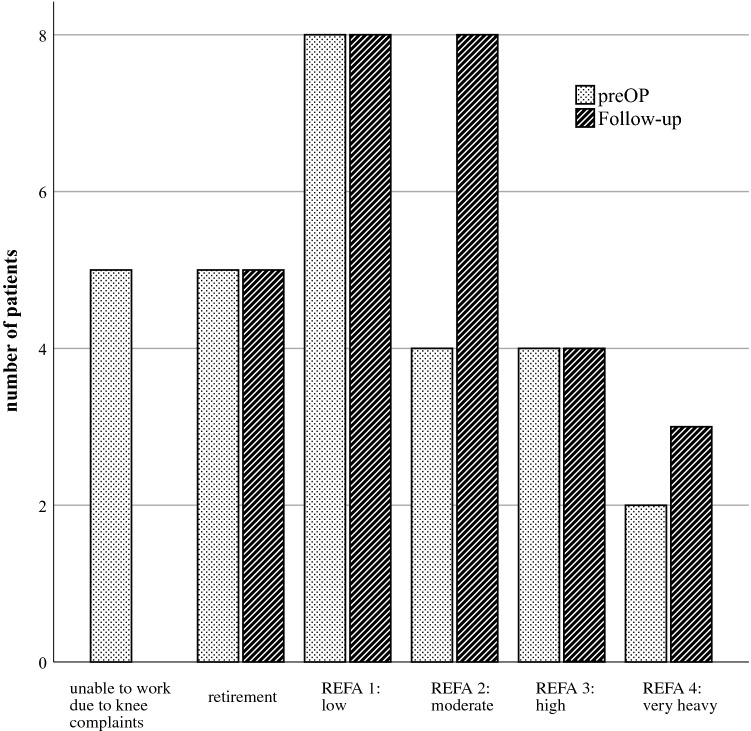
The work intensity measured with the REFA classification is presented preoperatively (preOP) and at the follow-up. Five patients were unable to work preoperatively due to knee complaints. At the follow-up, these 5 patients were able to work with moderate work intensity (4 patients) and even very heavy work intensity (1 patient). *preOP* preoperative recording, *REFA 1* low work intensity, *REFA 2* moderate work intensity, *REFA 3* high work intensity, *REFA 4* very heavy work intensity

Changes in work intensity were measured with the REFA classification and are presented in Fig. 4. Prior to surgery, 5 patients were already retired, and thus their data were not subjected to work-related analysis. All patients who were unable to work due to knee complaints (5 of 23 cases) returned to work in the short term. Four of these patients shifted to moderate and 1 to very heavy work intensity. The general duration of the inability to work was 12.2 ± 4.4 (6–20) weeks.

**Fig. 5:**
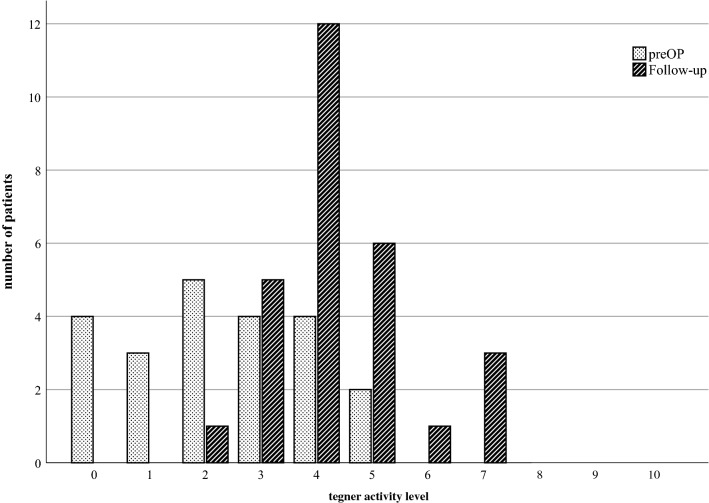
The Tegner activity scale increased between the preoperative period (preOp) and the follow-up examination

The Tegner activity scale increased significantly from a median of 2.0 (0.0–5.0) to 4.0 (2.0–7.0) (*p* < 0.001; Fig. 5). The VAS improved from 6.8 ± 2.3 (0.0–10.0) to 1.8 ± 1.9 (0.0–9.0) (*p* < 0.001).

## Discussion

The most important finding of the present study was that quality of life comparable to that of the general population at short-term follow-up can be achieved by DLO in patients with varus malalignment and medial compartment knee osteoarthritis. The physical aspects of the HRQL were reduced prior to surgery and improved at follow-up. All patients who were unable to work before surgery due to symptomatic varus knee osteoarthritis were able to return to work with moderate or even very heavy workloads. The duration of the inability to work was 12.2 ± 4.4 (6–20) weeks with an improved pain situation. Based on these results, surgeons can preoperatively assume that patients will return to work following DLO in the short term regardless of the work intensity. Moreover, patient HRQL will be improved, leading to high patient satisfaction. This information can be used to improve patient education, preoperative counselling and expectation management.

### Quality of life

The incidence of osteoarthritis increases with age, while quality of life decreases in the elderly population. This is based on several factors, such as a reduction in daily activities and increased physical disabilities and morbidities [25, 35]. Knee osteoarthritis has a substantial negative impact on both patients’ daily living activities and their expectations of treatment outcomes [4]. Subjective factors such as pain, social and physical role functioning and mental and emotional stability are often compromised in patients with degenerative joint diseases. These factors are important criteria for evaluating treatment outcomes. For example, Saier et al. showed that the preoperative mental health status and expectation of the treatment outcome of the patient are relevant to the clinical outcome after HTO [37]. Furthermore, quality of life is an important factor for returning to work [16, 37].

The results of the present study showed improvement in the quality of life in the short term, especially in its physical aspects, which is comparable to previously published literature on open wedge HTO surgery [15, 42]. For example, Herbst et al. described an improvement during the first 6 months in the physical components score of the SF-36 from 31.4 ± 14.0 preoperatively to 40.7 ± 14.5 [15]. However, the scores were lower than those of the general population (18.6 ± 14.0) preoperatively, after 6 months (9.3 ± 14.5) and after 12 months (4.9 ± 15.0). They [15] also showed that the mental components score of the SF-36 was similar preoperatively and 6 months postoperatively to that of the general population and increased by an average of 2.5 ± 12.1 points during the first 6 months. This is in accordance with the results of the present study. Herbst et al. also showed a slight decline in the quality of life between 18 and 72 months postoperatively following HTO [15]. Saier et al. reported that patients achieved the maximum subjective quality of life (SF-36) 6 to 12 months after open wedge HTO and that it remained largely constant [19]. Based on the short follow-up period of the present study, the mid- and long-term quality of life remains unclear and needs further investigation. In addition to improvement in quality of life following DLO, previous studies reported good clinical midterm outcomes [5, 22, 28]. DLO can restore lower limb alignment and joint angles by preventing changes in leg length in varus malalignment. Furthermore, joint line obliquity can be avoided compared to isolated high tibial osteotomy [3, 5, 39].

### Return to work

Especially for young patients, a high rate of return to work and a short duration of work inability are important criteria for the treatment decision to avoid work disadvantages even job loss due to a long absence. In the present study, all patients except those who were retired at the start of the study were able to work after DLO. This high rate of return to work is comparable to that following open wedge HTO, which has been found to be 85% on average, ranging between 41% and 100% [17]. Approximately nine out of ten patients treated with distal femoral osteotomy returned to work [8, 19, 36]. In addition to returning to the workplace, patients aim to reach the same work intensity they had preoperatively. As shown by Agarwalla et al. [1], young and active patients with medial compartment osteoarthritis are able to return to work with the same work intensity as prior to HTO surgery. This is in accordance with the data of the present study regarding DLO treatment, as patients with high work intensity were able to return to the same intensity as before. However, patients working in higher intensity occupations need more time to return to the workplace following osteotomies around the knees [1, 7, 9]. Puzzitiello et al. [36] showed that all 32 patients with valgus knee osteoarthritis treated with distal femoral varus osteotomy returned to work an average of 6 months postoperatively. The duration of work absence was dependent on the work intensity; patients with high work intensity needed 13.8 months, and patients with light work intensity needed an average of 2.7 months. Seven patients in their cohort changed jobs or kept the same job but had a lighter workload. Patients following HTO were unable to work on average 2.9 ± 2.0 months [2] or 87 days [38]. In the cohort of the present study, the mean duration of work inability was 12.2 ± 4.4 weeks, with a maximum of 20 weeks. This underlines that DFO, DLO or HTO treatment for knee osteoarthritis has similar effects regarding restoration of the patient’s work ability.

In addition to work intensity, other factors influence the ability to return to work, including socioeconomic factors such as disability coverage, health insurance and economic need [18]. Horrntje et al. [18] showed that breadwinners were more likely to return to work, whereas patients with preoperative sick leave were less likely to return to work within 6 months after HTO surgery. Furthermore, postoperative quality of life and return to work are dependent on the incidence of intraoperative and postoperative complications. The reported complications for DLO and their incidence are similar to those of open wedge HTO. Both the incidence of hinge fractures, of which 30.6% are related to the medial hinge in lateral closed wedge distal femoral osteotomy [29], and the incidence of deep vein thrombosis (6.8% [31]) are comparable to those in HTO. Furthermore, the duration to returning to work is influenced by the postoperative rehabilitation protocol. Partial weight-bearing allows patients with low work intensity to return to work earlier. Early weight-bearing [13] and 6-week partial weight-bearing [39] have been described for postoperative rehabilitation, which is similar to the rehabilitation protocol of the present study.

The limitations of the present study are the short follow-up period and the wide range of follow-up durations. It remains unclear whether the patients were able to maintain the same work intensity over a longer period of time. However, all patients were able to return to their previous work as well as their previously described work intensity, at least in the short term. Furthermore, the study provides data about the subjective quality of life and the return to work after DLO, which were previously missing in the literature. In addition, significant improvements and even values comparable to the normal population were found in the short-term follow-up for the quality of life at the time of the return to work. Another limitation is the retrospective design for assessing the quality of life measured at the time of follow-up to determine the preoperative health status. However, Lawson et al. described that this type of data collection is a valid way to estimate the baseline health status [24]. A further limitation is the relatively small sample size, that no power analysis was performed and that four patients received DLO on both knees, but the score assessment was performed for both knees at one time point. However, there was an interval of at least 1 year between the first and second surgery, and patients returned to work between procedures. Further research is needed to analyse the impact of physiological joint angles as achieved by DLO on quality of life over time and survival rate. Although similar clinical outcomes and survival rates to HTO were achieved, it should be noted that DLO is usually preceded by a more severe preoperative axial deformity than HTO.

## Conclusions

The results of the present study demonstrate improvement in the quality of life and return to working activity following DLO even in the short term. Both quality of life and work intensity can be improved by DLO in patients with varus malalignment and medial compartment osteoarthritis to the levels of the general population. This knowledge is important for realistic patient education prior to lower limb deformity correction.

## Abbreviations

HRQL: Health related quality of life
HTO: High tibial osteotomy
DFO: Distal femoral osteotomy
DLO: Double level osteotomy
mLDFA: Mechanical lateral distal femoral angle
MPTA: Medial proximal tibia angle
PF: Physical functioning
RP: Role physical
BP: Bodily pain
GH: General health
VT: Vitality
SF: Social functioning
RE: Role emotional
MH: Mental health
MCS: Mental component summary score
PCS: Physical component summary score
VAS: Visual analogue scale
TKA: Total knee arthroplasty
